# Verrucous carcinoma of the vulva: Patterns of care and treatment outcomes

**DOI:** 10.1002/cnr2.1591

**Published:** 2022-01-24

**Authors:** Sara M. Dryden, Leonid B. Reshko, Jeremy T. Gaskins, Scott R. Silva

**Affiliations:** ^1^ Department of Radiation Oncology University of Louisville Louisville Kentucky USA; ^2^ Department of Bioinformatics & Biostatistics University of Louisville Louisville Kentucky USA

**Keywords:** gynecologic cancer, National Cancer Database, verrucous, vulva

## Abstract

**Background:**

Verrucous vulvar carcinoma (VC) is an uncommon and distinct histologic subtype of squamous cell carcinoma (SCC). The available literature on VC is currently limited to case reports and small single institution studies.

**Aims:**

The goals of this study were to analyze data from the National Cancer Database (NCDB) to quantitate the incidence of VC and to investigate the effects of patient demographics, tumor characteristics, and treatment regimens on overall survival (OS) in women with verrucous vulvar carcinoma.

**Methods and results:**

Patients diagnosed with vulvar SCC or VC between the years of 2004 and 2016 were identified in the NCDB. OS was assessed with Kaplan–Meier curves and the log‐rank test. Construction of a Cox model compared survival after controlling for confounding variables. The reported incidence of SCC of the vulva has significantly increased since 2004 (*p* < .0001). In contrast, the incidence of VC has remained stable (*p* = .344) since 2004. Compared to SCC, VC was significantly more likely to be diagnosed in older women (*p* < .0001) and treated with surgery alone (*p* < .0001). However, on propensity score weighted analysis there was a trend toward improved 5‐year OS in women with VC compared to those with SCC (63.4% vs. 57.7%, *p* = .0794). Multivariable Cox survival analysis showed an improvement in OS in VC patients treated with both primary site and regional lymph node surgery compared to primary site surgery alone (adjusted hazard ratio [aHR] 0.67, 95% confidence interval [CI] 0.46–0.97, *p* = .0357).

**Conclusion:**

Verrucous carcinoma is more likely to present in older women. Regional lymph node surgery in addition to primary site surgery significantly improves OS in VC patients.

## 1 INTRODUCTION

Verrucous carcinoma (VC) of the vulva is a rare form of squamous cell carcinoma that accounts for approximately 1% of vulvar tumors.^1^, ^2^ VC was first classified as a form of squamous cell carcinoma by Ackerman in 1948 in a case of VC of the oral cavity.^3^ In 1963 Goethals et al. first suggested involvement of the female genital tract, and in 1966 Kraus and Perez‐Mesa reported the first two cases of vulvar verrucous carcinoma.^4^, ^5^ Grossly, verrucous carcinoma is fungating and cauliflower like. Histologically, VC commonly displays organized keratinocytes, acanthosis, parakeratosis, or orthokeratosis, with minimal cellular atypia.^6^, ^7^ Blunt invasion with bulbous rete ridges is a characteristic finding.^8^, ^9^ Unlike well‐differentiated SCC, there is no cytologic atypia, invasion by irregular‐shaped nests of carcinoma, or desmoplastic stromal response in VC.^10^ Verrucous carcinoma can be associated with local recurrences; however, distant metastases from VC are uncommon.^11^, ^12^ Most cases begin with a small wart that progressively enlarges over several months. Chief complaints at the time of initial presentation range from concern for abnormal lesions, itching, and discomfort, to hindrance of daily activities depending on the size and extent of local lesion involvement.

The incidence of invasive vulvar carcinoma in the United States has been increasing over the past three decades across all age groups of women, among all races, and in all geographic regions.^13^ Theories of increased immunosuppressed populations, miscoded cases, and environmental factors have been suggested to explain the changing incidence of vulvar carcinoma. Verrucous vulvar carcinoma is more commonly diagnosed in post‐menopausal women.^8^, ^14^ VC is a well‐differentiated form of vulvar SCC, with most cases unrelated to infection with human papilloma virus (HPV).^12^ While the relationship between VC and HPV has been debated,^9^, ^15^, ^16^ some studies have suggested that a portion of VC cases are related to HPV.^17^

Treatment of VC is centered around primary site surgery, with attention toward taking wide margins to prevent local recurrence.^18^ Since lymph node metastases are rare in VC, radical vulvectomy is sufficient in the majority of cases, and systemic lymph node dissection is generally not performed.^18^, ^19^ Some cases of VC may coexist with SCC, in which a lymphadenectomy would be warranted in addition to primary site surgery.^20^ In an extensive literature review by Liu et al., 15% (10/67) of cases of VC coexisted with well‐differentiated SCC.^2^ This evidence of coexisting histologies places an emphasis on the importance of thorough histologic evaluation to achieve an accurate and complete diagnosis. There have been some cases of patients receiving radiotherapy and subsequent transformation to SCC or anaplastic transformation.^19^, ^21^, ^22^, ^23^, ^24^ The current paradigm regarding treatment for VC is that wide local excision is associated with a favorable prognosis.

The purpose of this study was to perform an analysis of the National Cancer Database (NCDB) (1) to assess the incidence of verrucous vulvar carcinoma, (2) to compare patient, tumor, and treatment characteristics between VC and vulvar SCC, and (3) to analyze factors associated with OS in verrucous carcinoma of the vulva.

## 2 METHODS

### 2.1 Database

This study analyzed data from the National Cancer Database (NCDB). The NCDB includes about 70% of all cases of cancer diagnosed in the United States every year. Extensive demographic data as well as disease and treatment details are provided for each de‐identified patient. Increased numbers of institutions are participating in recording cancer cases with the NCDB. In 2008, 1430 Commission on Cancer (CoC) programs reported to the NCDB, but in 2020 nearly 1500 CoC programs were participating.^25^

### 2.2 Patient cohort

**FIGURE 1:**
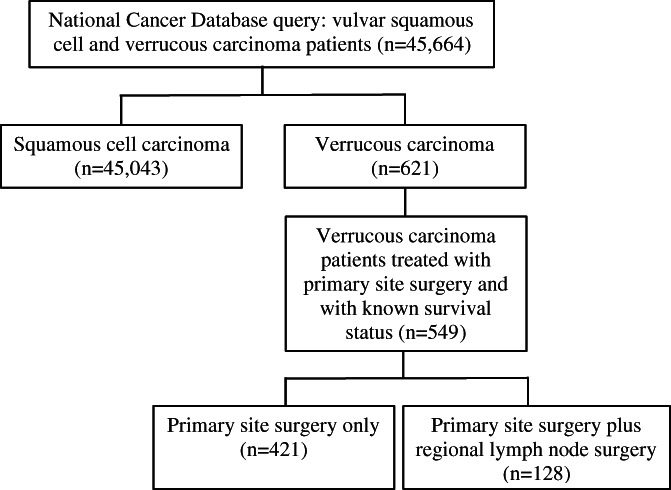
CONSORT diagram describing inclusion criteria

The NCDB included 621 patients diagnosed with VC and 45 043 patients diagnosed with SCC. Patients who were diagnosed with verrucous vulvar carcinoma or vulvar SCC between the years of 2004 and 2016 were included. The inclusion criteria for this analysis are shown in the CONSORT diagram in Figure 1.

### 2.3 Statistical analysis

Baseline patient demographics were obtained from the NCDB including age, race, tumor size and stage, surgical intervention, and adjuvant therapy. Statistical analysis was performed comparing cases of SCC to cases of VC by the chi‐squared test to evaluate differences in disease characteristics at diagnosis, surgical management, and adjuvant treatments. To evaluate changes in incidence across years, linear regression models were used to fit the logarithm of the number of cases as a function of year; a model using the ratio of VC to SCC cases was also considered to compare changes in the two histologies. The Kaplan–Meier method and log rank tests were performed to evaluate the differences in OS between VC and SCC and OS with regard to lymph node surgery in VC treatment. Patients diagnosed in 2016 had unknown survival status and were excluded from survival analyses. Propensity score weighted analysis was used to evaluate OS between VC and SCC while balancing confounding variables. A multivariable Cox proportional hazards model was utilized to assess the impact of various demographic factors and treatment methods on OS in the VC patients. All analyses were performed using the R project for statistical computing software, version 3.6.2. Statistical significance was defined by *α* < .05.

## 3 RESULTS

### 3.1 Incidence rates and demographics of VC compared to SCC

**FIGURE 2:**
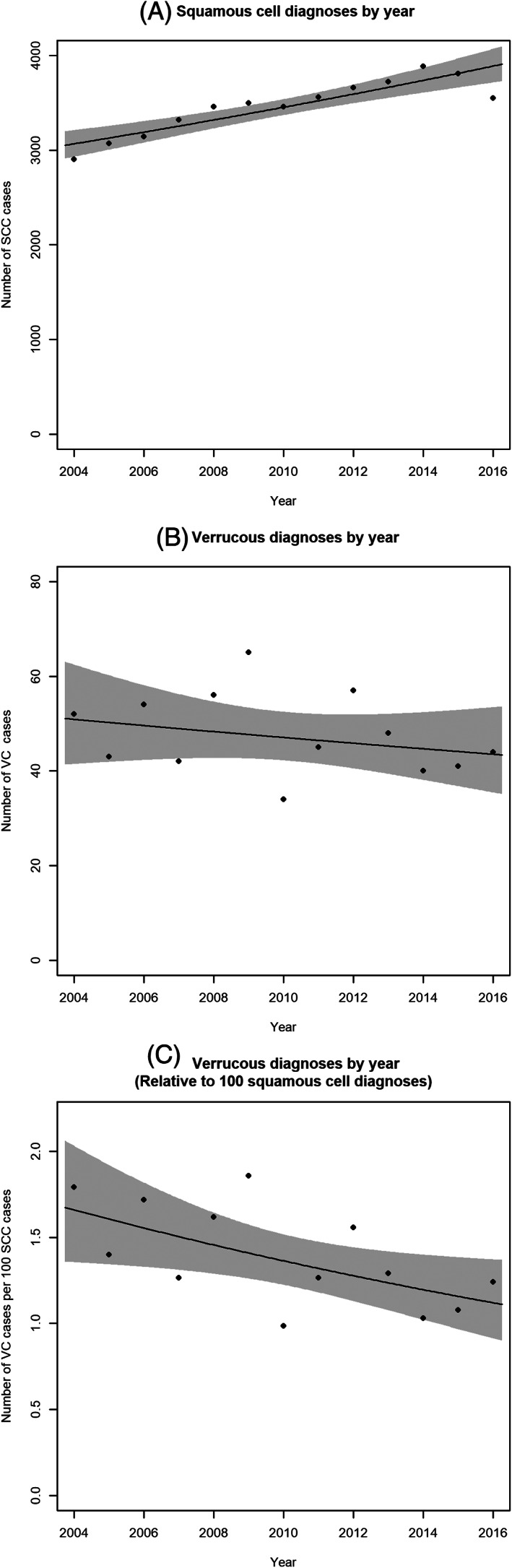
Incidence of vulvar cancer. (A) Squamous cell carcinoma. (B) Verrucous carcinoma. (C) Number of verrucous carcinomas per 100 squamous cell carcinoma cases

SCC diagnoses reported in the NCDB demonstrate an approximately 2.0% increase each year from 2004 to 2016 (CI 1.3% to 2.7%, *p* < .0001) as shown by Figure 2A. Conversely, the incidence of VC has remained stable, with diagnoses decreasing by a non‐significant 1.3% per year (CI 4.2% decrease to 1.6% increase; *p* = .344) from 2004 to 2016 as demonstrated in Figure 2B. To control for the potential effect of changing NCDB reporting rates, we considered the ratio of VC to SCC cases. As shown in Figure 2C, VC diagnoses are significantly declining by 3.2% per year relative to the number of SCC diagnoses (CI 0.4% to 6.0%; *p* = .0285).

A comparison of patient demographics between SCC and VC demonstrated many significant differences (Table 1). The age distribution between the two cohort subsets was statistically significantly different (*p* < .0001). Of all women diagnosed with SCC, 63.3% were under the age of 70. In contrast, 59.4% of women with VC were diagnosed at age 70 or over, and throughout the time period examined there was no change in the mean age of diagnosis for VC patients (*p* = .563). A significantly larger portion of the VC patients had a non‐zero Charlson/Deyo comorbidity score (VC: 34% vs. SCC: 27%, *p* < .0001). There were no significant differences between SCC and VC diagnoses based on race (*p* = .2044) or Hispanic origin (*p* = .4830).

**TABLE 1:** Cohort demographics comparing squamous cell carcinoma and verrucous vulvar carcinoma

	Squamous cell		Verrucous		
	*N*	Prop	*N*	Prop	*p*‐value
Histology	45 043	99%	621	1%	<.0001
Age					
<50	9872	22%	61	10%	
50–59	9743	22%	74	12%	
60–69	8919	20%	117	19%	
70–79	8030	18%	140	23%	
≥80	8479	19%	229	37%	
Race					.2044
White	39 843	88%	561	90%	
Black	3961	9%	42	7%	
Other	1239	3%	18	3%	
Hispanic origin					.4830
Non‐Hispanic	43 499	97%	596	96%	
Hispanic	1544	3%	25	4%	
Charlson/Deyo Comorbidity Score					<.0001
Absent	32 881	73%	409	66%	
Present	12 126	27%	212	34%	
Tumor Size					<.0001
< 2 cm	11 152	36%	122	25%	
2.0–3.9 cm	9169	30%	171	35%	
4.0–5.9 cm	4871	16%	93	19%	
≥ 6 cm	5393	18%	96	20%	
Unknown	14 458	139			
Grade					<.0001
1	10 157	34%	332	87%	
2	14 339	48%	40	10%	
≥ 3	5269	18%	11	3%	
Unknown	15 278	238			
TNM pathologic stage					<.0001
0	6261	21%	13	3%	
1	14 255	49%	236	63%	
2	3230	11%	98	26%	
3	4429	15%	20	5%	
4	1141	4%	10	3%	
Unknown	15 727	244			
Lymph vascular invasion					<.0001
No LVSI	15 668	89%	217	97%	
Invasion	1997	11%	6	3%	
Unknown	27 378	398			
Primary site surgery					<.0001
No surgery	6114	14%	28	5%	
Surgery	38 876	86%	593	95%	
Unknown	53	0			
Regional lymph node surgery					<.0001
No surgery	26 673	60%	482	78%	
Surgery	18 117	40%	138	22%	
Unknown	253	1			
Chemotherapy					<.0001
No chemo	37 582	86%	571	96%	
Chemo	6060	14%	24	4%	
Unknown	1401	26			
Radiotherapy					<.0001
No radiation	34 552	77%	572	93%	
Radiation	10 082	23%	40	7%	
Unknown	409	9			

Abbreviations: cm, centimeter; *N*, number; Prop, proportion, LVSI, lymph vascular invasion; TNM, tumor‐node‐metastasis.

### 3.2 Tumor characteristics and treatment modalities

Several tumor characteristics of SCC and VC were significantly different. Verrucous carcinomas tended to be slightly larger than squamous cell carcinomas (*p* < .0001), with 39% of verrucous carcinomas measuring at least 4 cm in size, compared to 34% of squamous cell carcinomas being 4 cm or larger. Tumor grade was significantly different (*p* < .0001) with the vast majority of VC (87%) being grade 1; in contrast, only 34% of SCC cases were grade 1. Comparison of TNM (tumor‐node‐metastasis) pathologic group staging between the two histologies also demonstrated a significant difference (*p* < .0001). The majority of VC cases were stage 1 or 2 (63% and 26%, respectively), with a limited number of stage 3 and 4 cases (5% and 3%, respectively). In contrast, a larger proportion of SCC patients were diagnosed with stage 3 or 4 disease (15% and 4%, respectively). Lymph vascular invasion was seen in a higher portion of SCC patients (11%) compared to VC patients (3%).

Treatment approaches were also significantly different between SCC and VC (*p* < .0001). Primary site surgery was done in a larger portion of VC patients (95%) compared to those with SCC (86%). Regional lymph node surgery was more often performed in patients with SCC (40%) in comparison to those with VC (22%). Adjuvant therapy such as chemotherapy or radiation therapy were more commonly used in cases of SCC. Chemotherapy was used more frequently in cases of SCC (14%) than VC (4%); similarly, radiotherapy was used in a much higher proportion of SCC (23%) compared to VC patients (7%).

### 3.3 Overall survival of SCC compared to VC

**FIGURE 3:**
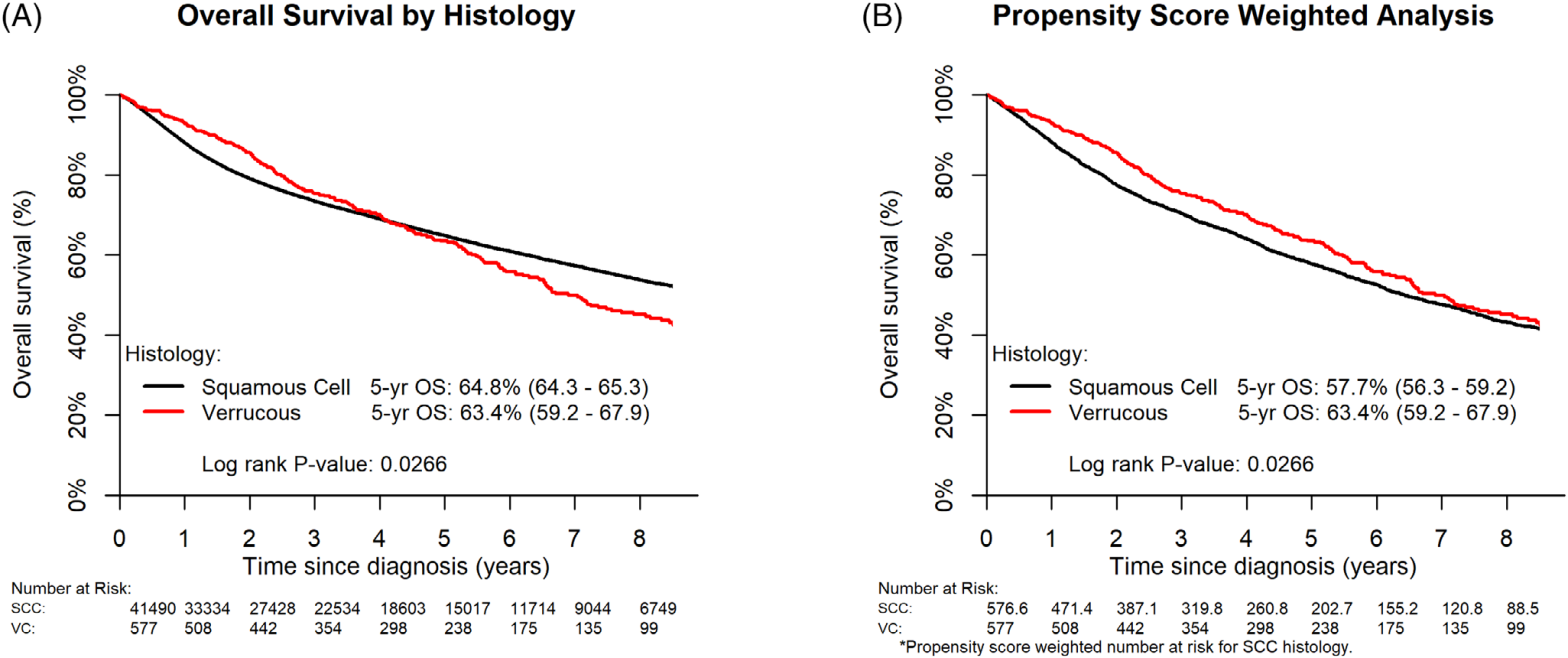
Overall survival by histology. (A) Kaplan Meier survival analysis. (B) Propensity score weighted analysis

There was a statistically significant difference in the Kaplan–Meier survival curves between patients diagnosed with squamous cell carcinoma compared to those diagnosed with verrucous vulvar carcinoma (*p* = 0.0266; Figure 3A). Five‐year survival rates where similar between the two histologies (SCC 64.8% and VC 63.4%). However, VC patients had statistically better survival soon after diagnosis (between years 1 and 2.5) but worse survival past year 7. Propensity score weighted analysis, which reweighted the SCC cohort to match the characteristics of the VC patients, demonstrated a trend toward improved OS in VC patients (*p* = 0.0794; Figure 3B).

### 3.4 Factors affecting OS in VC patients

A Cox proportional hazards analysis was performed to evaluate factors affecting OS in patients diagnosed with verrucous carcinoma (Table 2). Overall survival was significantly worse in VC patients who were older and in those who had a non‐zero Charlson/Deyo comorbidity score. Compared to patients younger than 50 years, those between the ages of 70–79 years had significantly worse OS (adjusted hazard ratio [aHR] 4.65; CI 2.22–9.75; *p* < .0001), as did patients ≥80 years (aHR 8.96; CI 4.35–18.45; *p* < .0001). A non‐zero Charlson/Deyo comorbidity score was associated with an aHR of 1.68 (CI 1.28–2.20; *p* = .0002). Regarding tumor characteristics, patients with at least grade 3 tumors also had significantly worse OS compared to those with grade 1 tumors (aHR 3.88; CI 1.57–9.57; *p* = .0032). Additionally, patients with pathologic stage 4 VC had significantly worse OS compared to VC patients with stage 1 disease (aHR 28.7; CI 3.37–244.07; *p* = .0021).

**TABLE 2:** Cox proportional hazards model for OS in patients with verrucous vulvar carcinoma

	Adj HR	95% CI		*p*‐values	
Age (years)				<.0001	
<50	Reference				
50–59	1.56	0.68	3.61		.2965
60–69	2.56	1.18	5.53		.0168
70–79	4.65	2.22	9.75		<.0001
≥80	8.96	4.35	18.45		<.0001
Race				.2000	
White	Reference				
Black	1.72	0.99	3.00		.0564
Other	1.06	0.43	2.65		.8963
Hispanic origin				.7256	
Non‐Hispanic	Reference				
Hispanic	0.88	0.41	1.86		.7299
Charlson/Deyo comorbidity score				.0002	
Absent	Reference				
Present	1.68	1.28	2.20		.0002
Tumor size				.5760	
<2 cm	Reference				
2.0–3.9 cm	1.18	0.80	1.76		.4036
4.0–5.9 cm	1.42	0.92	2.18		.1135
≥6 cm	1.24	0.79	1.96		.3491
Unknown	1.32	0.84	2.07		.2321
Grade				.0550	
1	Reference				
2	1.33	0.81	2.20		.2572
≥3	3.88	1.57	9.57		.0032
Unknown	1.10	0.83	1.46		.5151
TNM pathologic stage				.0006	
0	Reference				
1	3.04	0.41	22.78		.2783
2	4.30	0.56	32.77		.1591
3	3.07	0.37	25.71		.3010
4	28.70	3.37	244.07		.0021
Unknown	3.32	0.45	24.68		.2404
Lymph vascular invasion				.6294	
No LVSI	Reference				
Invasion	0.49	0.07	3.70		.4894
Unknown	1.11	0.73	1.70		.6209
Primary site surgery				<.0001	
No surgery	Reference				
Surgery	0.19	0.11	0.33		<.0001
Regional lymph node surgery				.0341	
No surgery	Reference				
Surgery	0.67	0.47	0.96		.0298
Unknown	0.00	0.00	Inf		.0034
Chemotherapy				.1252	
No chemo	Reference				
Chemo	0.44	0.19	1.02		.0560
Unknown	1.15	0.52	2.55		.7290
Radiotherapy				.0121	
No radiation	Reference				
Radiation	1.90	1.04	3.47		.0360
Unknown	0.21	0.04	1.09		.0636
Diagnosis year				.3865	
HR per year	1.03	0.97	1.09		.3865

Abbreviations: 95% CI, 95% confidence interval; Adj HR, adjusted hazard ratio; cm, centimeter; HR, hazard ratio; LVSI, lymph vascular invasion; OS, overall survival; TNM, tumor‐node‐metastasis.

**FIGURE 4:**
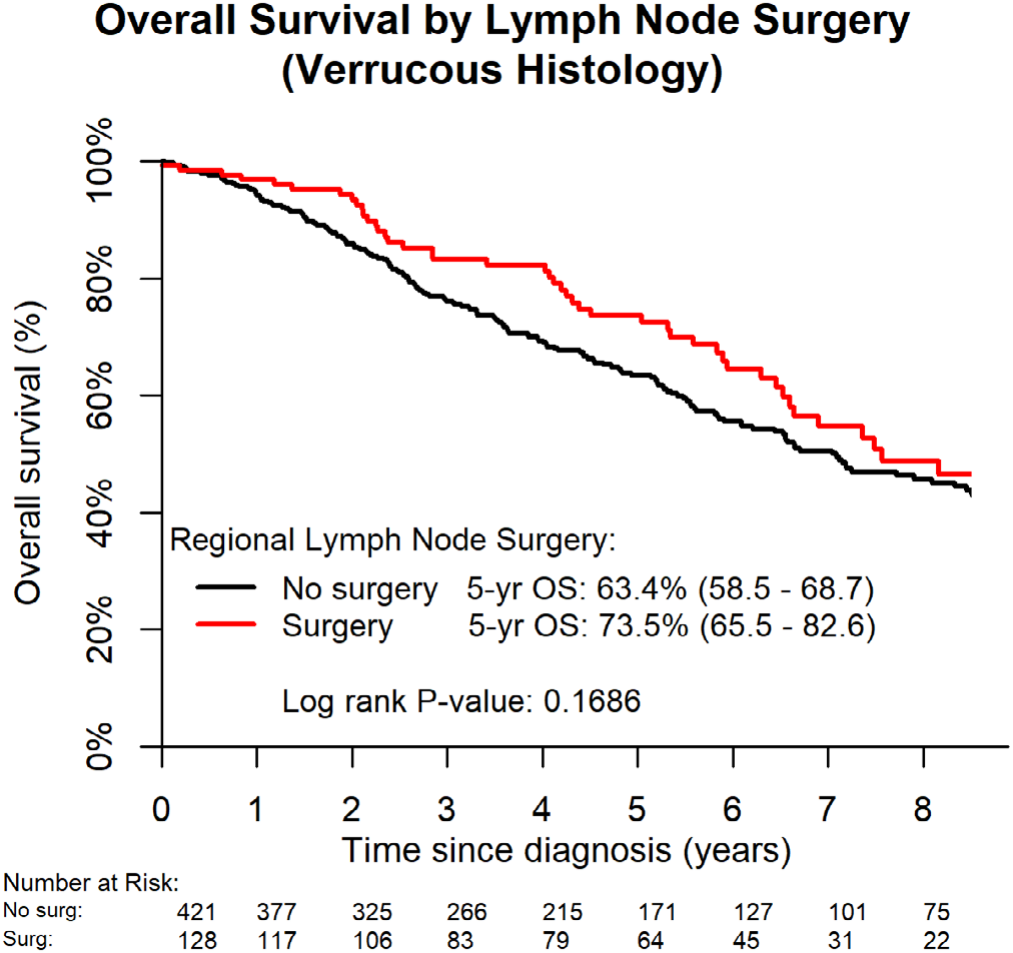
Overall survival by lymph node surgery

Different treatment modalities also had a significant effect on OS in VC patients (Table 2). Patients treated with surgery to the primary site had a significant improvement in OS compared to those with no primary site surgery (aHR 0.19; CI 0.11–0.33; *p* < .0001). On univariate analysis, there was a trend in improved 5‐year OS in VC patients treated with regional lymph node surgery plus surgery at the primary site compared to primary site surgery alone (73.5% vs. 63.4%, respectively, *p* = .1686) (Figure 4). Cox proportional hazards analysis demonstrated a significant improvement in OS in women who were treated with surgery to the regional lymph nodes (aHR 0.67; CI 0.47–0.96; *p* = .0298) when controlling for other confounders. When excluding VC patients who were not treated with surgery to the primary site, the combination of primary site surgery plus regional lymph node surgery also improved OS compared to treatment with primary site surgery alone on multivariate analysis (aHR 0.67; CI 0.46–0.97; *p* = .0357). Patients who received chemotherapy had a trend toward improved OS (aHR 0.44; CI 0.19–1.02; *p* = .0560), while VC patients who received radiotherapy had significantly worse OS (aHR 1.90; CI 1.04–3.47; *p* = .0360).

## 4 DISCUSSION

### 4.1 Incidence rates of verrucous vulvar carcinoma

The incidence of vulvar SCC has significantly increased from 2004 to 2016. This trend could be due to the increase in risk factors throughout the population. Cigarette smoking, BMI > 30, and menopausal hormones have all been associated with an increased risk of vulvar SCC.^26^ The number of VC cases diagnosed annually is trending down, but this could be impacted by differing levels of NCDB case capture between 2004 to 2016. In particular, NCDB reporting rates have increased which could mask a true decrease in VC incidence if more recent cases are reported at a higher frequency. Relative to the number of SCC cases reported in the NCDB, VC cases are significantly declining.

### 4.2 Patient demographics

Most studies have concluded that VC is diagnosed in patients at a later age than SCC,^2^, ^27^, ^28^ and our results are consistent with this observation. While there have been case reports of VC patients diagnosed at a younger age,^29^ our results do not support a trend toward a decreasing age of diagnosis of verrucous vulvar carcinoma.

### 4.3 Differing tumor characteristics and treatment modalities

Regional lymph node surgery was performed more commonly in SCC cases, which is expected since lymph node metastases rarely occur in VC.^18^, ^19^ Lymphadenectomy is generally recommended with a primary tumor infiltration depth > 1 mm.^30^ Verrucous carcinoma is rarely infiltrative, which may account for the decreased rate of lymph node surgery. In our analysis, primary site surgical intervention was performed in the vast majority of VC, and results showed that a smaller proportion of SCC cases received surgery (85% SCC, 95% VC). The observed high rate of primary site surgery in VC cases is likely because wide local excision with adequate margins is the current standard of care for VC.^18^ Furthermore, because VC is more often diagnosed at an earlier stage than SCC, complete resection of the primary tumor without performing an excessively morbid surgery (i.e., pelvic exenteration) is more likely in cases of VC.

Chemotherapy and radiotherapy were used more frequently in SCC compared to VC, with only 4% and 7% of VC patients receiving chemotherapy or radiotherapy, respectively. This is most likely due to the increased propensity of SCC to metastasize to regional and distant sites compared to VC.^11^, ^12^ Additionally, radiotherapy in VC treatment has been associated with anaplastic transformation and possible progression to invasive SCC.^19^, ^21^, ^22^, ^23^, ^24^

### 4.4 Limitations

We acknowledge limitations to this study. First, large population‐based data sets are prone to missing and inadequate data. Rather than excluding patients with missing covariates from our overall survival model, we simply treated “unknown” as a distinct category so that all patients were included in the analysis. For completeness, we did choose to continue to report these values since they were a part of the model fitting. In general, we are not confident that the data are actually missing at random. Many of the aHRs for missing values were greater than 1 indicating missing data may be associated with greater hazard of death, although these terms are not always significant. We suspect that missing values may be correlated with poorer patient care which may then be correlated with poorer survival, but to avoid making any explicit assumptions, we have chosen to treat missing values as its own category. Additionally, the NCDB only lists one histologic diagnosis per patient, and, thus, an evaluation of patients with a coexistence of VC and SCC could not be performed. Second, the vast majority of VC patients are diagnosed at an early stage and grade, and these patients are overwhelmingly treated with surgery without chemotherapy or radiotherapy. Thus, any statistical comparison of VC based on stage, grade, and treatment other than surgery should be interpreted with caution. Third, a study performed in 2017 analyzed the accuracy of histology code reporting in central cancer registries, and subtypes of vulvar SCC were not consistently recorded with proper histologic codes when compared to the pathology reports.^31^ These findings expose a limitation in the study of rare subtypes of SCC, including verrucous vulvar carcinoma.

### 4.5 Conclusions

While the incidence of vulvar SCC has increased since 2004, the incidence of VC has remained stable. Compared to SCC, VC was significantly more likely to be diagnosed in older women and treated with surgery alone. We found that there was an improvement in OS in VC patients treated with both primary site and regional lymph node surgery compared to primary site surgery alone.

## CONFLICT OF INTEREST

The authors have stated explicitly that there are no conflicts of interest in connection with this article.

## AUTHOR CONTRIBUTIONS

*Conceptualization*, S.M.D., L.B.R., and S.R.S.; *Methodology*, J.T.G.; *Formal analysis*, S.M.D., L.B.R., S.R.S., and J.T.G.; *Critical analysis*, S.M.D., L.B.R., S.R.S., and J.T.G.; *Statistical analysis*, J.T.G; *Drafting/final editing*, S.M.D., L.B.R., S.R.S., and J.T.G.; *Supervision*, S.R.S.

## ETHICS STATEMENT

The study was found to be exempt from Institutional Review Board (IRB) review through 45 CFR 46.116 (D) by our institution's Biomedical Institutional Review Board.

## Data Availability

The data that supports the findings of this study are available in the National Cancer Database.
